# Tracking adaptation strategies of an HIV prevention intervention among youth in Nigeria: a theoretically informed case study analysis of the 4 Youth by Youth Project

**DOI:** 10.1186/s43058-023-00404-8

**Published:** 2023-04-26

**Authors:** Juliet Iwelunmor, Oliver Ezechi, Chisom Obiezu-Umeh, Titilola Gbaja-Biamila, Adesola Z. Musa, Ucheoma Nwaozuru, Nnamdi Obasi, Victor Ojo, Hong Xian, David Oladele, Collins O. Airhihenbuwa, Kathryn Muessig, Nora Rosenberg, Donaldson F. Conserve, Jason J. Ong, Susan Nkengasong, Kadija M. Tahlil, Rhonda BeLue, Alexis Engelhart, Stacey Mason, Weiming Tang, Gbenga Ogedegbe, Joseph D. Tucker

**Affiliations:** 1 College for Public Health & Social Justice, Saint Louis University, 3545 Lafayette Avenue, Saint Louis, MO 63104 USA; 2 Clinical Sciences Department, Nigerian Institute of Medical Research, Lagos, Nigeria; 3 Department of Implementation Science, Division of Public Health Sciences, Wake Forest School of Medicine, Winston-Salem, NC USA; 4 College of Medicine, University of Nigeria, Nsukka, Nigeria; 5 Federal University of Technology Akure, Akure, Ondo State Nigeria; 6 Heath Policy and Behavioral Sciences, School of Public Health, Georgia State University, Atlanta, GA USA; 7 Gillings School of Global Public Health, University of North Carolina at Chapel Hill, Chapel Hill, NC USA; 8 Department of Prevention and Community Health, Milken Institute School of Public Health, The George Washington University, Washington, DC USA; 9 Central Clinical School, Monash University, Melbourne, Australia; 10 Melbourne Sexual Health Centre, Alfred Health, Melbourne, Australia; 11 Clinical Research Department, London, School of Hygiene and Tropical Medicine, London, UK; 12 Community and Policy, College for Health, University of Texas at San Antonio, San Antonio, TX USA; 13 Department of Medicine, University of North Carolina at Chapel Hill, Chapel Hill, NC USA; 14 Center for Healthful Behavior Change, Division of Health and Behavior, Department of Population Health, New York University School of Medicine, NY, New York, NY USA; 15 Faculty of Infectious and Tropical Diseases, London School of Hygiene and Tropical Medicine, London, UK

**Keywords:** HIV, Adolescent, Young adult, Adaptation, Implementation science, Sub-Saharan Africa

## Abstract

**Background:**

Although many behavioral interventions are adapted, little is known about the reasons for adaptations and the process and outcomes influencing adaptations. To address this gap, we explored the adaptations made to promote HIV prevention services, including HIV self-testing (HIVST), among Nigerian youth.

**Methods:**

The main objective of this qualitative case study design was to document the adaptations made over time using the Framework for Reporting Adaptations and Modifications – Expanded (FRAME). Between 2018 and 2020, we organized four participatory activities as part of the 4 Youth by Youth project to increase the uptake of HIVST services in Nigeria—an open call, a designathon, a capacity-building bootcamp and a pilot feasibility trial. We also began the process of implementing a final intervention using a pragmatic randomized control trial (RCT). The open call solicited creative strategies to promote HIVST among Nigerian youth and then had experts evaluate them. The designathon brought together youth teams to further develop their HIVST service strategies into implementation protocols. Teams determined to be exceptional were invited to a four-week capacity-building bootcamp. The five teams that emerged from the bootcamp were supported to pilot their HIVST service strategies over a 6-month period. The adapted intervention is currently being evaluated in a pragmatic RCT. We transcribed meeting reports and conducted document reviews of study protocols and training manuals.

**Results:**

Sixteen adaptations were identified and categorized into three domains: (1) modifications to the content of the intervention (i.e. photo verification system and/or Unstructured Supplementary Service Data (USSD) system to verify HIVST); (2) modifications to the delivery the intervention (i.e. implement participatory learning community sessions to provide supportive supervision and technical support); (3) modifications to the evaluation processes (i.e. economic evaluation to estimate the cost of implementing intervention on a larger scale). Frequent reasons for adaptation included increasing intervention reach, modifying interventions to enhance their appropriateness and fit with the recipient, and increasing the intervention’s feasibility and acceptability. Most adaptations were planned and reactive, and the need for modifications was determined by the youths, 4YBY program staff, and advisory group.

**Conclusions:**

Findings suggest that the nature of adaptations made throughout the implementation process reflects the necessity of evaluating services in context while adjusting to specific challenges as they are identified. Further research is needed to understand the effect of these adaptations on the overall intervention effect as well as the quality of youth engagement.

**Supplementary Information:**

The online version contains supplementary material available at 10.1186/s43058-023-00404-8.

Contributions to the literature

1. We used FRAME to advance the science of adaptation among youth populations traditionally under-represented in the implementation literature.
2. We expanded the literature by providing a practical examination of why, what, and how adaptations occurred within an HIV prevention intervention focused on youth.
3. We provide how-to-do-it literature on standardized reporting of adaptation processes used to refine HIV prevention interventions in a low- and middle-income country context.

## Background

The use of evidence-based HIV interventions for adolescents and young people living in Nigeria is suboptimal. Despite numerous triumphs in HIV, adolescent and young adult (AYA; aged 10–24 years old) contributions to HIV prevention research activities has been limited [1]. As a result, tremendous gaps remain along the HIV prevention continuum for AYA at risk [1, 2]. In Nigeria, adolescents are at significant HIV risk, but very few are tested, and many do not have access to high quality youth-friendly prevention services [3, 4]. For example, in a study with over 14,000 AYAs in Nigeria, less than a quarter (23.7%) had ever tested for HIV and a lower proportion (12.4%) tested in the year prior to administering the survey [5]. Further, most Nigerian youths have an early sexual debut [6] (median age at first sexual activity is 16 for females and 17 for males) and high-risk sexual behaviors. Previous studies have revealed that early sexual initiation is one of the most robust predictors of sexually transmitted infections (STI) among AYAs [7]. HIV/STI testing is critical both as the gateway to HIV/STI treatment, which can lead to reduced onward transmission and as a critical first step to access prevention strategies such as pre-exposure prophylaxis (PrEP) [8, 9]. To stop HIV/STIs among AYA, it is essential to routinely engage youth in the uptake of evidence-based prevention strategies to not only maximize the public health benefits of these interventions but also to enhance fit with the contexts in which they are implemented.

Conventional evidence-based interventions for AYA are often top-down, expert-driven one-size-fits-all processes, with few opportunities for input from AYA themselves [10–12]. This is problematic as failure to allow the perspectives of AYAs whether in developing, vetting, or implementing HIV interventions often results in programs that do not resonate or address the needs and priorities of AYAs [11, 13]. Yet, to maximize the adoption, use and sustainability of these interventions in real-world settings among youth populations, deliberate alterations of designs, or delivery may need to occur to improve the fit of interventions in a given context and with youth populations [14, 15]. The process of modifying interventions and/or implementation strategies to better fit a new context or population is known as adaptation, as explained by Stirman and colleagues [16]. Among young people, understanding adaptation to interventions may be beneficial for the following reasons. First, adaptations allow researchers and young people to exchange, generate, and utilize collaborative knowledge that may enhance the impact of evidence-based HIV prevention services [17]. Second, adaptations may facilitate not only the adoption of evidence-based interventions but also facilitate the sustainment of implementation, which is the integration of an innovation or intervention within a specific setting or context, over time [17]. Moreover, Chambers and colleagues [15, 18] suggested that optimizing interventions through adaptations is more likely to enhance the sustainment of the evidence-based intervention defined as programs, practices, innovations, policies, and guidelines with proven effectiveness and efficacy [19]. Third, studies suggest that adaptations may improve appropriateness or fit between the intervention and population/context [20, 21].

Nonetheless, despite the benefits and/or challenges with adaptation, little is known about real-world adaptations of interventions whether planned or unplanned as part of implementing evidence-based HIV interventions. As a result, and because many evidence-based interventions proven to be effective are never sustained over time in low and middle-income country settings [22], this study advances knowledge on the science of adaptation in the context of implementing HIV prevention services among youth populations traditionally under-represented in implementation research. The purpose of this study is to explore the reasons, process and outcomes of adaptations made to an ongoing pragmatic randomized control trial of crowdsourced interventions to expand HIV/STI testing among youth in Nigeria. The overall goals were to describe the adaptations made during the pre-implementation, implementation, and post-implementation phases. Findings will contribute to the literature on adaptations using widely recognized and standardized reporting tools such as the Framework for Reporting Adaptations and Modifications Expanded tool.

## Methods

### Implementation setting and population

Beginning in 2018 and in collaboration with the Nigerian Institute for Medical Research (NIMR), we designed and implemented the Innovative Tools to Expand Youth-friendly HIV Self-Testing (I-TEST) project (known locally as 4 Youth by Youth (4YBY). We combined evidence-based open calls, designathons, apprenticeship training, and pilot testing to expand the reach and uptake of evidence-based oral HIV self-testing (HIVST) among adolescents and youth aged 14–24 years in Nigeria [23]. Crowdsourcing open calls have a group of individuals attempt to solve a problem and then share their solutions with the public [24–27]. Once participants are engaged, an apprenticeship strategy pairs them with local experts for advice-seeking as well as to further build capacity for developing and implementing new youth-friendly HIV self-testing services [28]. Apprenticeship provides the practical skills, direct mentorship, and supportive environment to increase the likelihood of launching successful HIV self-testing services targeting youth populations [28]. Pilot testing of newly developed HIVST services in real-time provides youth designers/implementers with real-world opportunities to facilitate the adoption of their HIVST services, as well as exchange experiences with implementation barriers and/or facilitators. The finalist crowdsourced HIVST intervention was implemented among youth 14–24 years in Nigeria. Additional details on the study protocol can be found in a separate publication [29].

### Participatory intervention development

**Fig. 1:**
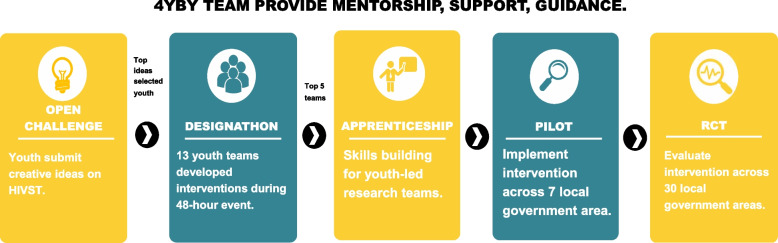
Participatory intervention development

Guided by the youth participatory action research (YPAR) framework, the process followed five steps in which through engaging young people in the design and adaptation of solutions to better meet their needs, youth-friendly HIVST service strategies iteratively emerged (Fig. 1). Prior to the intervention development procedures, we convened a youth advisory board, organizing committee and judging team, each of which included Nigerian youths, professionals/researchers in public health, communications, civil society, and product design. The youth advisory board and organizing committee worked collaboratively to oversee the day-to-day activities of the open call process, establishing open call rules and promoting the open calls. First, we organized a crowdsourcing open call from October to December 2018 to solicit concepts and strategies to promote HIVST among AYAs in Nigeria [23]. The open call was designed following standardized approaches to crowdsourcing as recommended by the WHO Special Programme for Research and Training in Tropical Diseases practical guide [30]. The call for entries was disseminated online on various social media platforms and in person within local communities. Following multiple steps of screening (for relevance to the open call and plagiarism) and evaluation (based on its desirability to young people, feasibility to implement, and potential for impact with an integer score of 1 (low) to 3 (high) in each domain), people who submitted top strategies were invited to participate at a designathon in teams of 2–5 individuals. The finalist strategies informed a 48-h designathon in which 13 teams of AYA were invited through an open call to participate and design prototypes to launch their HIVST services [31]. Each team, in turn, finalized and presented their prototypes to an expert panel of judges. The expert panel consisted of Nigerian professionals in public health, communications, civil society, product design, and youth ambassadorship. The judges evaluated the proposals based on desirability to young people, feasibility to implement, and potential for impact, on a three-point scale with one being a low score, two being a medium score and three being a high score. The five best scoring teams from the designathon participated in a 4-week apprenticeship training, which we titled Innovation Training Bootcamp, where they received mentorship, and information on HIVST, including how to encourage and facilitate linkage to facility-based HIV confirmatory testing and STI testing services [32]. Additionally, their strategies were further refined and finalized to increase uptake and reach at-risk youth. In collaboration with NIMR, the five teams pilot tested their HIVST packages across seven local government areas in Nigeria from September 2019 to March 2020. Lastly, based on the pilot data as well as feedback from the AYA participants [33], we then incorporated key components of the five HIVST service packages into a combination intervention strategy that is now being evaluated in a pragmatic, stepped-wedge, cluster randomized controlled trial across 32 local government areas in Nigeria. The resulting intervention included (1) HIVST bundle consisting of HIVST kits and photo verification and and/or the Unstructured Supplementary Service Data (USSD) system to verify the HIVST result and facilitate linkage to STI services; (2) a participatory learning community to identify and resolve implementation challenges real-time; (3) peer to peer support and technical assistance from the youth advisory board as well as the local study team; and (4) on-site supervision and performance feedback to improve uptake and sustainability of HIVST and enhance linkage to youth-friendly health clinics for confirmatory HIV testing where needed, sexually transmitted infection (STI) testing (i.e. syphilis, gonorrhea, chlamydia, and hepatitis, STI treatment, and PrEP referral [34].

### Stakeholder engagement

**Fig. 2:**
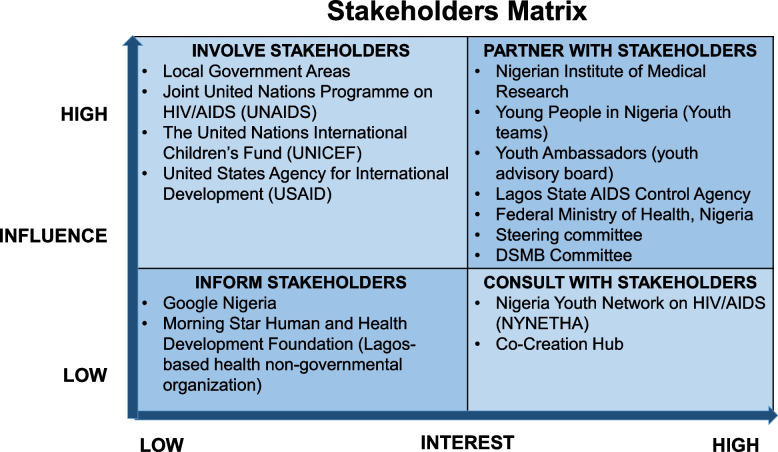
4 Youth by Youth stakeholders by level of interest and influence

Stakeholder engagement is essential for adapting interventions. Prior to the intervention development process, we mapped out key stakeholders based on their stated positions, level of influence they held, the level of their interest with addressing the HIV prevention continuum among AYA, and their group affiliations. Based on these factors, we created a stakeholder matrix (Fig. 2) based on their degree of influence and level of interest and support towards HIV services for adolescents and young adults in Nigeria. Some of the key stakeholders included the Nigeria Federal Ministry of Health, local government area leaders, the Nigerian AIDS Control Agency, the Lagos State AIDS Control Agency, Community Alliances (i.e. Nigeria Youth Network on HIV/AIDS—a youth coalition to promote HIV prevention and management in Nigeria), and 4YBY youth ambassadors, (a youth advisory board who provide input and feedback on all research activities to ensure that they are appropriate and acceptable for Nigerian youths), and Co-creation Hub for the development of the HIV self-testing mobile phone verification application. The identification and involvement of these stakeholders helped to foster ownership and fit of the youth-led HIV self-testing interventions to the local context. In addition to national and local agencies, we identified and engaged with several international organizations that work in Nigeria (i.e., UNAIDS, UNICEF, Google Nigeria) as key stakeholders of the research. Finally, and leveraging existing research resources in Nigeria, our local implementers are the Nigerian Institute of Medical Research (NIMR), the apex research establishment in the country. NIMR is responsible for leading and coordinating all implementation, thus enhancing the opportunities for the continuation of the intervention components overtime. These stakeholders together formed the 4YBY advisory group. We used face-to-face meetings at different phases of the study with the advisory group to discuss ways to enhance intervention implementation for youth populations in Nigeria.

### Data collection

We conducted a qualitative case study approach, as defined by Stake [35], which provides a flexible way of examining the complexities of a specific case in a real-world setting.

We leveraged formative data collected from 2018 to 2020 during the crowdsourcing events and pilot implementation as well as with efforts to optimize and implement the final crowdsourced HIV/STI intervention. We also conducted document reviews of study protocols, six training manuals, and transcribed forty-eight meeting reports to assess factors motivating intervention implementation at different phases, including changes made with intervention implementation throughout the life cycle of the study. Additional information on the reasons for implementation and considerations for maintaining the interventions overtime were also explored [36].

### Framework for implementation

**Fig. 3:**
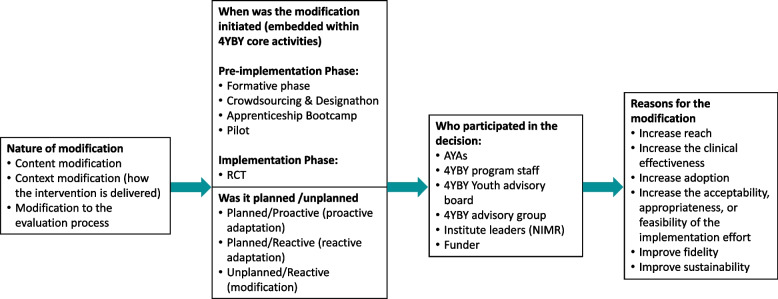
Adapted FRAME model used to describe modifications to 4YBY intervention

The Framework for Reporting Adaptations and Modifications Expanded (FRAME) [16, 17] was used as a guide to document our adaptation process. FRAME is a comprehensive framework that enables documentation of adaptations made to an intervention and/or implementation strategy, and it includes the following (Fig. 3): (1) when during the implementation process the modifications were made; (2) whether the modifications were planned or unplanned and proactive or reactive; (3) the individuals involved in the decision to modify program elements; and (4) the nature of context (e.g., format, setting) and content modifications (e.g., tailored, substituted, removed elements). Information on intervention and implementation strategy adaptations were collected through document review (i.e. protocol changes), stakeholder interviews, and data collected from youth participants as part of the crowdsourcing, pilot phase, and in preparation of the ongoing stepped-wedge cluster randomized control trial.

### Data analysis

We used the immersion crystallization process to analyze all the data generated [37]. An immersion crystallization process allows for a reflection of text generated until insights and interpretations are reached. We used a three-step process to complete this process to identify adaptations overtime. First, two researchers (NO and VO), who were working under the supervision of the lead researcher (JI), independently read all available documents and data to generate a list of I-TEST adaptations from the beginning and overtime until no new adaptations are identified. Triangulation of data sources was conducted by using the multiple sources to ensure that the adaptations were adequately captured. An audit trail of the coding process was maintained. Second, a research team made up of principal investigators, youth researchers, and study team members who led the intervention during crowdsourcing, pilot, and RCT phase reviewed and discussed as a group; the preliminary list was generated by the reviewers to create a final list of adaptations. Third, using the final identified list, two raters from the research team used FRAME as a guide to rating the adaptations made over time (Fig. 3). Disagreements on the classification of adaptations with FRAME were resolved through consensus among the research team.

## Results

### Characteristics of modifications

A total of sixteen modifications were identified using FRAME as a guide. Majority of the adaptations occurred during the pre-implementation phase of the study (75%) and were planned and reactive (63%) to aspects of intervention implementation overtime. Program staff made majority of the modifications overtime (100%) with input generated from stakeholder advisory groups including youth teams themselves. Most of the modifications also focused on increasing intervention reach (50%) among youth populations overtime.

**Fig. 4:**
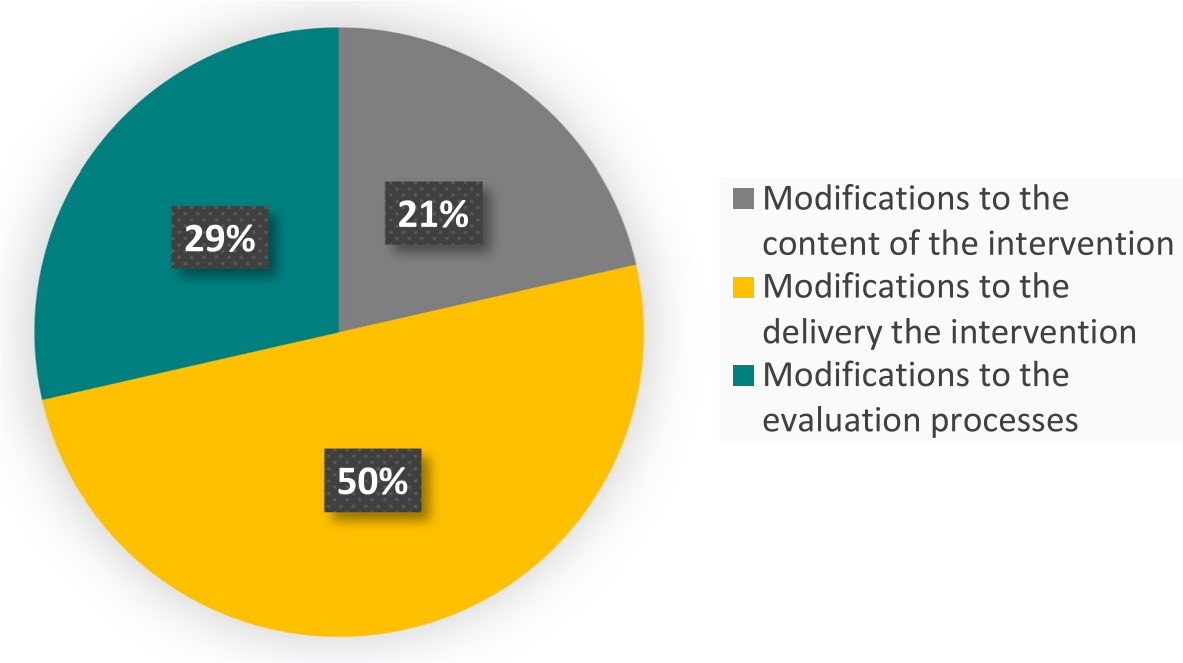
Nature of modifications

All sixteen adaptations were categorized into three main domains informed by categories in FRAME (Fig. 4): (1) modifications to the content of the intervention; (2) modifications to the delivery the intervention; (3) modifications to the evaluation processes. Table 1 provides an overview of the adaptations including what elements were adapted, how, when, and why the adaptation occurred. Most notably, some adaptations were in response to the 4YBY advisory board, 4YBY program staff, including the youth advisory board members and youth implementing teams, whereas other adaptations were in response to the funders and the institution in which the program was situated.

**Table 1:** Adaptations (*n* = 16) made to intervention coded according to FRAME

Nature of modification	Stage of intervention development	Description	Description of adaptation	When is the modification initiated, and is it planned	Who participated in decision to modify	Reasons for modification
**Modifications to the content of the intervention**	Crowdsourcing and designathon	Integrate game features such that participants will earn points based on actions they take (i.e., voting, commenting and submitting new ideas) to optimize engagement	No game features were used. However, we optimized engagement through our social media platforms and youth ambassadors	Pre-implementation; planned/reactive	4YBY program staff particularly the YAB members	Increase feasibility and appropriateness
	Crowdsourcing and designathon	Implement a crowdsourcing and designathon contest	Continuous crowdsourcing open contests on World AIDS Day (December 1) each year	Implementation phase; unplanned reactive	4YBY advisory group; 4YBY program staff	Improve sustainability
	Pilot	Conduct community ranking exercise online (one IP address per vote) and at one in-person event in each of the areas	We did not implement the community ranking exercise as this was not feasible	Pre-implementation phase; unplanned/reactive	4YBY program staff	Improve appropriateness
	Pilot	HIVST results will be self-reported by the study participants via a self-administered questionnaire	HIVST results will be confirmed through a photo verification system and/or Unstructured Supplementary Service Data (USSD) system	Pre-implementation phase; planned pro-active	Funders; 4YBY advisory group; 4YBY program staff particularly the YAB members;4YBY youth teams	Increase fidelity
	Pilot	Utilize wallet-size appointment cards at enrollment to monitor rates of return/retention	We utilized barcoded referral coupons to facilitate linkage to facility-based STI testing and maximize retention	Pre-implementation phase; planned reactive	4YBY program staff; 4YBY youth teams	Increase acceptability and appropriateness
**Modifications to the delivery the intervention**	Formative phase	The formative stage of discrete choice experiments (DCE) will require a sample size of 30 participants for in-depth interviews	We conducted 65 qualitative in-depth interviews to inform the DCE survey development	Pre-implementation; planned/reactive	4YBY program staff	Increase reach and appropriateness
	Apprenticeship bootcamp	Organize a 6-week apprenticeship bootcamp to build entrepreneurial capacity for HIVST startups to implement their plans	We shortened the duration of the apprenticeship bootcamp 4-weeks long	Pre-implementation phase; planned/reactive	4YBY advisory group; collaboration between 4YBY program staff and youth participants	Increase feasibility and appropriateness
	Pilot	Implement two semi-finalist participatory interventions	Five semi-finalist teams were supported to implement five distinct participatory interventions (i.e., pilot studies)	Pre-implementation phase; unplanned/reactive	4YBY program staff and local key stakeholders	Increase adoption
		Recruit a total of 280 youth in four local government areas (LGA) to participate in two pilot studies	A total of 387 youth were enrolled within seven LGAs across the five pilot studies	Pre-implementation phase; unplanned/reactive	4YBY advisory group; 4YBY program staff	Increase reach and improve effectiveness
		Progress reports will be provided to a Youth Advisory Board (YAB) on quarterly basis	We engaged the YAB beyond providing them with monthly updates. The YAB in collaboration with the program staff collaborated to provide peer support and technical assistance to the youth implementers throughout the series of participatory learning community sessions	Pre-implementation phase; planned/reactive	4YBY program staff particularly the YAB members	Improve fidelity
	RCT	Implement the finalist intervention across 24 LGAs in a stepped wedge, pragmatic randomized controlled trial	The finalist intervention is implemented across 32 LGAs	Implementation phase; Unplanned reactive	4YBY program staff	Increase reach; improve effectiveness; increase health equity
		Group sites to transition six at a time from the control condition to the participatory intervention in a stepped wedge, pragmatic randomized controlled trial	Sites were grouped to transition eight at a time	Implementation phase; unplanned reactive	4YBY program staff	Increase feasibility
**Modifications to the evaluation processes**	Formative phase	Through DCEs, select youth will be asked whether they prefer attributes of the two semi-finalist interventions or conventional attributes developed by public health authorities	The DCE was conducted to identify which components of HIV testing and self-testing are most appealing to Nigerian youth	Pre-implementation phase; planned reactive	4YBY advisory group; 4YBY program and institutional staff	Increase acceptability and appropriateness
	Pilot	Implement an anonymous survey to determine youth preferences for the final participatory intervention	We included measures to assess overall youth participation quality and experience	Pre-implementation phase; Planned proactive	4YBY program staff	Increase acceptability
		Assess PrEP Initiation outcome	Due to the limited availability of PrEP in the country, we assessed PrEP knowledge and awareness as an alternative outcome	Pre-implementation phase; unplanned reactive	4YBY advisory group; 4YBY program staff	Increase feasibility
	RCT	Determine per unit costs associated with the intervention	We are conducting a robust cost-effectiveness analysis to estimate the cost per client of HIV prevention services delivered	Implementation phase; planned reactive	4YBY advisory group; 4YBY program staff and institutional staff	Improve sustainability

### Domain 1: Modifications to the content of the intervention

**Fig. 5:**
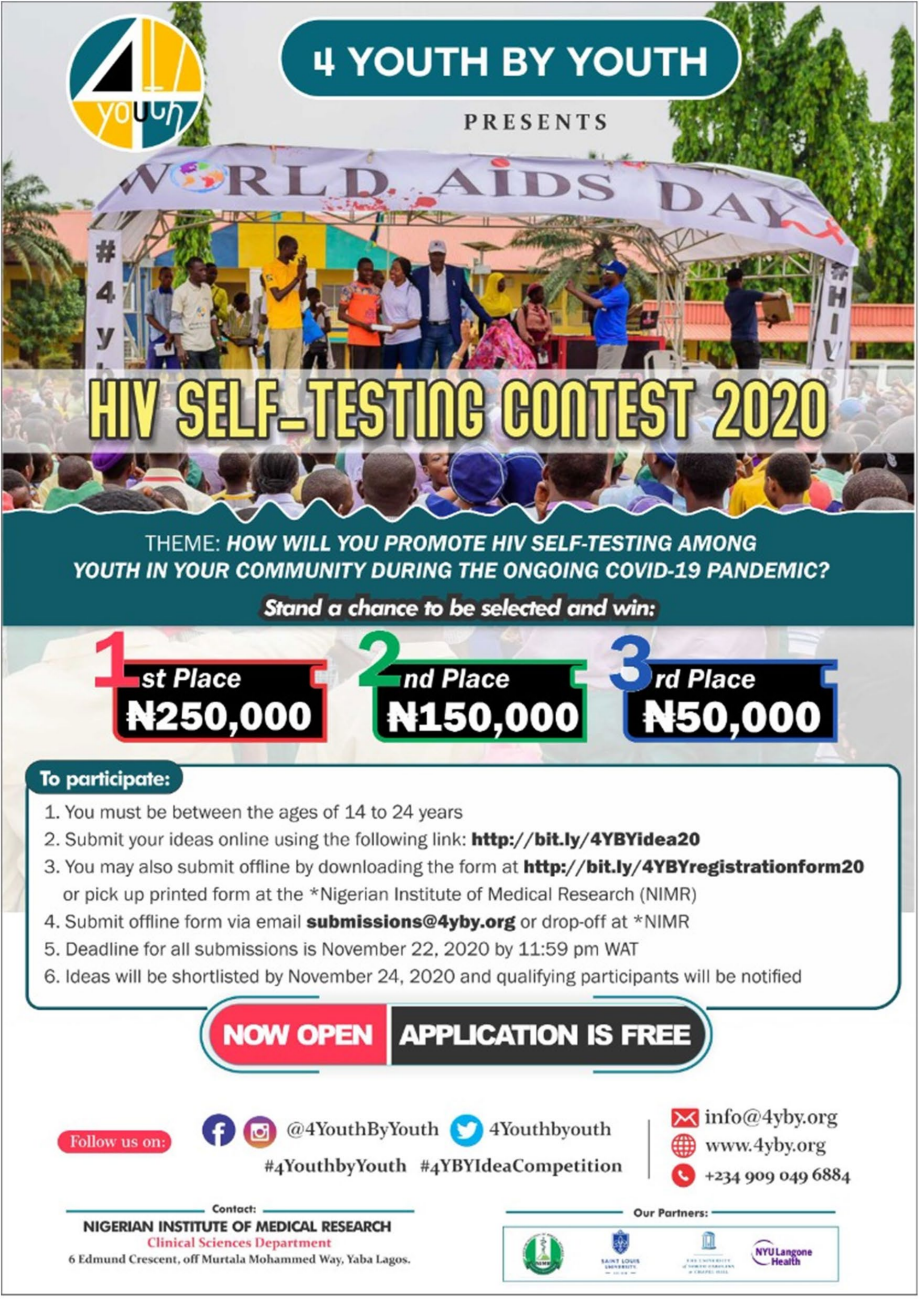
4 Youth by Youth (4YBY) crowdsourcing open contests on World AIDS Day

During the pre-implementation phase, most modifications to the content involved adding, tailoring, or removing intervention elements. Additionally, the content modifications were driven by the need to increase the feasibility, appropriateness, acceptability, fidelity, and sustainability of the intervention. To optimize youth participation and engagement during the crowdsourcing and designathon contest, the 4YBY program staff, as well as the youth advisory board members, recommended engaging youth through a combination of approaches, including in-person community-based events, social media messages. Many Nigerian AYA did not have sufficient bandwidth or smartphones needed for more complex gamification features, which was originally proposed by the youth as a way of enhancing engagement throughout the study. As a result, the 4YBY advisory group and as well as the program staff developed new USSD technologies that could be used to engage youth in low bandwidth settings. To sustain the delivery of the HIVST services while addressing potential implementation barriers, we co-organized the crowdsourcing open contest each year on World AIDS Day in collaboration with the Nigerian Institute of Medical Research (NIMR), state-level and nation-level AIDS control Agency (Fig. 5). Additional steps were undertaken during the pilot and RCT phase to improve the feasibility of the intervention and increase the retention of the study participants. These adaptations included removing the community ranking exercise to determine youth preferences for the final participatory Intervention and utilizing photo verification or the Unstructured Supplementary Service Data (USSD) system to verify HIVST results and linkage to care. Following an HIVST, participants were provided with two options to verify test results. The phone verification mobile phone application required a smartphone and allowed individuals to take a photograph of a completed test kit and send the encrypted message to study staff. Meanwhile, the USSD system can be used with analog phones and in settings with limited bandwidth. Following the HIVST, youth participants received information on the nearest youth-friendly clinic for follow-up testing and support. Additionally, the youth implementers utilized barcoded referral coupons to monitor linkage to facility-based STI testing as well as participant retention.

### Domain 2: Modifications to the delivery of the intervention

Several context adaptations were made including adaptations to the intervention setting, the delivery format, and the focus population. For instance, following the crowdsourcing and designathon phase, the program staff, as well as the youth teams, recognized the need to modify the format of the bootcamp from 6 to 4 weeks in order to accommodate conflicting demands faced by the youth teams. Context changes during pilot implementation involved expanding the original primary audience of the intervention as well as study sites, to increase the reach of the intervention while improving service delivery. The 4YBY advisory group and as well as the program staff suggested including additional study sites during the pilot implementation to ensure good representation of youth from various ethnicities, urban, rural, and remote communities across Nigeria. Similarly, in the RCT phase of the study, additional study sites were included to account for two other sites that were terminated due to civil unrest in some regions of the country. Prior to implementation, the youth identified several service delivery barriers related to traditional HTS and HIVST such as provider attitudes and linkages to appropriate care and support following HIVST. As such, the Youth Advisory Board (YAB) provided peer support and technical assistance to the youth implementers to ensure that they follow program procedures and identify and address implementation challenges in real-time. In line with this, each study site received supportive supervision through a series of participatory learning community (PLC) sessions [38]. The PLC was organized around the Plan-Do-Study-Act (PDSA) cycle [39–41], wherein the youth teams, YAB, and program staff collaborated to identify problems and barriers to implementation, define a strategy that can produce change, test the strategy, and use locally generated data to determine and compare across sites if the change yielded a process improvement.

### Domain 3: Modifications to evaluation processes

Modification related to the intervention evaluation process or outcomes often involved the selection of alternative methods or additional outcome measures so that the effect of new intervention components and approaches can be assessed. In the formative phase, the program staff originally planned to use the discrete choice experiments (DCE) to assess the two semi-finalist interventions that emerged from the apprenticeship bootcamp against conventional HIV testing services. However, the DCE was conducted concurrently with the crowdsourcing activities to identify HIV testing and self-testing service configurations as well as components of services that are most appealing to different groups of Nigerian youth. Findings from this study informed the development of the HIVST service packages during the designathon and apprenticeship bootcamp. For example, based on the findings from the DCE, majority of the youth indicated a preference to pay a small fee of up to 500 Naira ($USD1.38) to access the HIVST services. This small fee was incorporated into the HIVST service models to ensure the sustainability of HIV testing programs overtime. The program staff included additional secondary outcome measures to assess the overall youth participation experience during the pilot implementation, including the extent to which youth were substantially engaged and valued throughout the intervention process. The pre-exposure prophylaxis (PrEP) initiation outcome measure was modified upon the realization that PrEP services were not widely available in the country at the time of the study. To estimate the potential impact of PrEP availability, data on PrEP awareness, attitudes, willingness, and HIV-risk practices were collected. Although no formal cost analysis was planned initially, we embedded cost-effectiveness analysis as part of the study aims for the RCT phase to estimate how limited resources can best be deployed to maximize HIV prevention impact among Nigerian youth.

## Discussion

Given the limited information on the content (i.e. what) and processes (i.e. how) of adapting interventions targeting the youth population, we used FRAME to understand and characterize the reasons (i.e. why) for modifying an HIV prevention intervention targeting young people in Nigeria [16, 17]. The 4 Youth by Youth program made extensive planned and reactive adaptions ranging from modifications to the content of the interventions to changes to its delivery as well as changes to evaluation processes guiding the interventions [16]. Content adaptations were primarily at the level of youth themselves and generally involved adding program components to increase reach and retention of young people in HIV prevention interventions. Delivery adaptations mostly targeted the organizational context in which the interventions were being implemented as well as youth populations. These adaptations included changes to align the intervention to the needs of young people, and changes to increase engagement, not just with youth populations themselves but also with an external youth advisory group who were instrumental in providing peer support and technical assistance to youth teams. Finally, evaluation adaptations helped to align the intervention within the broader ecological context associated with implementing interventions in resource-limited settings, paying attention to costs, youth participation, and overall satisfaction with interventions, while also adapting to limited HIV prevention services within Nigeria such as with the availability of PrEP.

Our findings are similar to prior studies on adapting interventions in other settings and with youth populations. In a systematic review of adaptations of evidence-based public health interventions globally, the most common combinations of adaptations focused on the content, context, and delivery of the interventions [21]. Nearly all of the interventions described modifying interventions to fit new intervention populations, with changes made to the content and delivery of the original intervention [21]. Other benefits of adaptation included active involvement of key stakeholders to ensure that materials are culturally compelling and relevant to the target audience [21]. Likewise, and using FRAME as a guide, we expanded the literature to include planned versus unplanned adaptations as well as patterns of modification made with the 4YBY program. We also reported reasons for adaptations the types of modifications made within the program and by whom and when to enable other practitioners to learn from this process. Given that much work remains to understand the role adaptations may play in increasing reach, adoption, and ultimately sustainment of evidence-based HIV prevention interventions, our findings reflect the complex and dynamic settings in which implementation targeting youth populations occur.

Nonetheless, through an iterative process of multidimensional stakeholder engagement, we developed a flexible and nimble adaptation process to the 4YBY HIV prevention package with relevance to a broad range of partners, including youth populations themselves. The key success to our adaptation process was including our target population, young people themselves, whether as part of the youth advisory board or as youth teams developing and implementing the intervention packages. Young people as partners and not merely passive recipients of 4YBY [42] were critical in helping us understand how different approaches to implementing HIV prevention could enhance intervention reach and ultimately adoption among their peers [10, 43, 44]. This is particularly critical in reaching youth populations who face complex individual, social, and structural barriers to accessing interventions in general, including HIV prevention services [44, 45]. Our adaptation process also benefited greatly from incorporating an advisory group of key stakeholders who were pivotal in understanding how local leaders could use the results of the study to further enhance the uptake of HIV prevention services among youth populations in Nigeria. The advisory group provided additional perspectives, particularly on costing HIV prevention interventions that would be crucial and valuable in achieving the national goals for an AIDS-free generation in Nigeria by 2030. These findings underscore the need for further involvement of relevant stakeholders, including young people in the process of making adaptations to HIV prevention interventions. Although no ethical dilemmas were observed with participating in the overall 4YBY intervention development and implementation process given the age of consent in research participation at 14 in Nigeria [46], future studies may explore the shared challenges of minor adolescent consent to HIV research participation (particularly among youth aged 10–13), to identify strategies that would be feasible and acceptable with incorporating their perspectives in these contexts [47].

## Limitations

Our study has limitations worth mentioning. Most of the data utilized in this paper were through document reviews and self-reported data on intervention implementation and satisfaction. Although we utilized two raters and used FRAME as a guide to increase the reliability of the data abstracted, the potential for social desirability existed given that the raters had intimate knowledge of 4YBY core program activities. Future research on the best methods to document adaptations may enhance ways to describe and code adaptations based on valid and reliable detailed reporting standards for program adaptation that advance the field. Nonetheless, an additional limitation is that we did not assess the impact of the adaptation on implementation and behavioral outcomes. Future studies will focus on how these adaptations subsequently impacted outcomes such as intervention adoption or reach.

## Sustainability

After the first open call and subsequent activities leading to the implementation of finalist interventions, simultaneously from year 2, we continued to implement crowdsourcing events that gathered youth perspectives on HIV prevention services. We continue to work with diverse stakeholders who have been instrumental in identifying low-cost strategies and windows of opportunities such as the annual World AIDS Day event to sustain the 4YBY intervention activities overtime. We also created a sustainability plan [22] that includes people or key stakeholders to continue to collaborate with, learning from them, adapting where necessary, and nurturing existing resources overtime, to realize the vision of the continued use of intervention components for the continued achievement of desirable health outcomes among young people in Nigeria.

## Implications

The findings have implications for understanding not only the depth and extent of adaptations but ways to ensure that they reflect the unique needs of target populations and context. Future studies should report the role socio-political context may play in implementing interventions with youth populations in Nigeria, paying attention to how adaptations at the organizational, provider, and recipient factors may increase reach and engagement and enhance retention while addressing cultural factors that improve fit, feasibility, and sustainability of HIV prevention interventions targeting youth populations.

## Conclusion

This paper offers a practical examination of the adaptation process of an HIV prevention intervention targeting youth populations in Nigeria. With adaptations occurring naturally in settings, FRAME provided a framework for examining not only the reasons, but also the content, and processes associated with the 4YBY HIV prevention services in Nigeria [17]. It also facilitated an understanding of factors that may enhance transferability, while increasing the scale-up and spread of HIV interventions to achieve population impact [21]. Overall, findings underscore the need to increase reporting on the reasons, content, and process by which adaptations are made to enhance the reach, adoption, and ultimately sustainment of evidence-based HIV prevention interventions targeting youth populations. Understanding how interventions are adapted to increase reach or improve the fit between interventions and contexts [15] may go a long way towards inform strategies for adaptation within the context of implementing evidence-based HIV prevention interventions targeting youth populations.

## Supplementary Information

**Additional file 1.** Submission explanation - case studies in global ISR1.

## Data Availability

All data generated or analyzed during this study are included in this published article.

## Abbreviations

4YBY: 4 Youth By Youth
AYA: Adolescent and young adults
HIV: Human immunodeficiency virus
HIVST: HIV self-testing
HTS: HIV testing services
I-TEST: Innovative Tools to Expand Youth-friendly HIV Self-Testing
LGA: Local government area
NIMR: Nigerian Institute for Medical Research
PATC^3^H: Prevention and Treatment through a Comprehensive Care Continuum for HIV-affected Adolescents in Resource Constrained Settings
PDSA: Plan-Do-Study-Act
PrEP: Pre-exposure prophylaxis
SSA: Sub-Saharan African
STI: Sexually transmitted infections
USSD: Unstructured Supplementary Service Data
YPAR: Youth participatory action research

## References

[1] Ogundare EO, Olatunya OS, Oluwayemi IO, Inubile AJ, Taiwo AB, Agaja OT (2017). Pattern and outcome of dog bite injuries among children in Ado-Ekiti. Southwest Nigeria Pan Afr Med J.

[2] Rosenberg NE, Obiezu-Umeh CS, Gbaja-Biamila T, Tahlil KM, Nwaozuru U, Oladele D (2021). Strategies for enhancing uptake of HIV self-testing among Nigerian youths: a descriptive analysis of the 4YouthByYouth crowdsourcing contest. BMJ innovations..

[3] NDHS (2013). National Population Commision; Nigerian Demographic and Health Survey Reports.

[4] NACA (2015). Federal Republic of Nigeria, Global AIDS Report: Country Progress Report: Nigerian Agency for the Control of AIDS.

[5] Ajayi AI, Awopegba OE, Adeagbo OA, Ushie BA (2020). Low coverage of HIV testing among adolescents and young adults in Nigeria: implication for achieving the UNAIDS first 95. PLoS ONE.

[6] Odimegwu C, Somefun OD (2017). Ethnicity, gender and risky sexual behaviour among Nigerian youth: an alternative explanation. Reprod Health.

[7] Epstein M, Bailey JA, Manhart LE, Hill KG, Hawkins JD, Haggerty KP (2014). Understanding the link between early sexual initiation and later sexually transmitted infection: test and replication in two longitudinal studies. J Adolesc Health.

[8] Obiezu-Umeh C, Gbajabiamila T, Ezechi O, Nwaozuru U, Ong JJ, Idigbe I (2021). Young people’s preferences for HIV self-testing services in Nigeria: a qualitative analysis. BMC Public Health.

[9] Kwan TH, Lee SS (2018). Predictors of HIV testing and their influence on PrEP acceptance in men who have sex with men: a cross-sectional study. AIDS Behav.

[10] Asuquo SE, Tahlil KM, Muessig KE, Conserve DF, Igbokwe MA, Chima KP (2021). Youth engagement in HIV prevention intervention research in sub-Saharan Africa: a scoping review. J Int AIDS Soc.

[11] Oliveras C, Cluver L, Bernays S, Armstrong A (2018). Nothing about us without RIGHTS—meaningful engagement of children and youth: from research prioritization to clinical trials, implementation science, and policy. J Acquir Immune Defic Syndr..

[12] Tucker JD, Tang W, Li H, Liu C, Fu R, Tang S, et al. Crowdsourcing designathon: a new model for multisectoral collaboration. BMJ Innovations. 2018;4(2):46–50.

[13] Bekker L-G, Siberry GK, Hirnschall G (2018). Ensuring children and adolescents are not left behind. J Acquir Immune Defic Syndr..

[14] Cabassa LJ, Baumann AA (2013). A two-way street: bridging implementation science and cultural adaptations of mental health treatments. Implement Sci.

[15] Chambers DA, Norton WE (2016). The adaptome: advancing the science of intervention adaptation. Am J Prev Med.

[16] WiltseyStirman S, Baumann AA, Miller CJ (2019). The FRAME: an expanded framework for reporting adaptations and modifications to evidence-based interventions. Implement Sci.

[17] Miller CJ, Barnett ML, Baumann AA, Gutner CA, Wiltsey-Stirman S (2021). The FRAME-IS: a framework for documenting modifications to implementation strategies in healthcare. Implement Sci.

[18] Chambers DA, Glasgow RE, Stange KC (2013). The dynamic sustainability framework: addressing the paradox of sustainment amid ongoing change. Implement Sci.

[19] Rabin BA, Brownson RC, Haire-Joshu D, Kreuter MW, Weaver NL (2008). A glossary for dissemination and implementation research in health. J Public Health Manag Pract.

[20] Chlebowski C, Hurwich-Reiss E, Wright B, Brookman-Frazee L (2020). Using stakeholder perspectives to guide systematic adaptation of an autism mental health intervention for Latinx families: A qualitative study. J Community Psychol.

[21] Escoffery C, Lebow-Skelley E, Haardoerfer R, Boing E, Udelson H, Wood R (2018). A systematic review of adaptations of evidence-based public health interventions globally. Implement Sci.

[22] Iwelunmor J, Blackstone S, Veira D, Nwaozuru U, Airhihenbuwa C, Munodawafa D (2015). Toward the sustainability of health interventions implemented in sub-Saharan Africa: a systematic review and conceptual framework. Implement Sci.

[23] Iwelunmor J, Ezechi O, Obiezu-Umeh C, Gbaja-Biamila T, Nwaozuru U, Oladele D (2020). The 4 youth by youth HIV self-testing crowdsourcing contest: a qualitative evaluation. PLoS ONE.

[24] Tucker JD (2017). Crowdsourcing to promote HIV testing among MSM in China: study protocol for a stepped wedge randomized controlled trial. Trials.

[25] Tang W, Han L, Best J, Zhang Y, Mollan K, Kim J (2016). Crowdsourcing HIV testing: a pragmatic, non-inferiority randomized controlled trial in China. Clin Infect Dis.

[26] Zhang Y, Kim JA, Liu F, Tso LS, Tang W, Wei C (2015). Creative contributory contests to spur innovation in sexual health: 2 cases and a guide for implementation. Sex Transm Dis.

[27] Day S, Li C, Hlatshwako TG, Abu-Hijleh F, Han L, Deitelzweig C (2021). Assessment of a crowdsourcing open call for approaches to university community engagement and strategic planning during COVID-19. JAMA Netw Open.

[28] Billett S (2016). Apprenticeship as a mode of learning and model of education. Educ Training.

[29] Iwelunmor J, Tucker JD, Obiezu-Umeh C, Gbaja-Biamila T, Oladele D, Nwaozuru U (2022). The 4 Youth by Youth (4YBY) pragmatic trial to enhance HIV self-testing uptake and sustainability: study protocol in Nigeria. Contemp Clin Trials.

[30] WHO/TDR/SESH/SIHI (2018). Crowdsourcing in health and health research: a practical guide.

[31] Tahlil KM, Obiezu-Umeh C, Gbajabiamila T, Nwaozuru U, Oladele D, Musa AZ (2021). A designathon to co-create community-driven HIV self-testing services for Nigerian youth: findings from a participatory event. BMC Infect Dis.

[32] Nwaozuru U, Gbajabiamila T, Obiezu-Umeh C, Uzoaru F, Mason S, Tahlil K (2020). An innovation bootcamp model to develop HIV self-testing social enterprise among young people in Nigeria: a youth participatory design approach. Lancet Glob Health.

[33] Iwelunmor J, Ezechi O, Obiezu-Umeh C, Gbaja-Biamila T, Musa AZ, Nwaozuru U (2022). Enhancing HIV self-testing among Nigerian youth: feasibility and preliminary efficacy of the 4 Youth by Youth study using crowdsourced youth-led strategies. AIDS Patient Care STDS.

[34] Iwelunmor J, Tucker JD, Obiezu-Umeh C, Gbaja-Biamila T, Oladele D, Nwaozuru U, et al. The 4 Youth by Youth (4YBY) pragmatic trial to enhance HIV self-testing uptake and sustainability: study protocol in Nigeria. Contemp Clin Trials. 2021;114:106628. 10.1016/j.cct.2021.106628 PMC9358609 34800699

[35] Stake RE (2005). Qualitative case studies. The Sage handbook of qualitative research.

[36] Iwelunmor J, Tucker JD, Ezechi O, Nwaozuru U, Obiezu-Umeh C, Gbaja-Biamila T, Airhihenbuwa CO. Sustaining HIV Research in Resource-Limited Settings Using PLAN (People, Learning, Adapting, Nurturing): Evidence from the 4 Youth by Youth Project in Nigeria. Curr HIV/AIDS Rep. 2023;1–10. 10.1007/s11904-023-00652-2 PMC10102056 36988831

[37] Miller WL, Crabtree BF (1994). Qualitative analysis: how to begin making sense. Fam Pract Res J.

[38] Nix M, McNamara P, Genevro J, Vargas N, Mistry K, Fournier A (2018). Learning collaboratives: insights and a new taxonomy from AHRQ’s two decades of experience. Health Aff.

[39] Steinmo SH, Michie S, Fuller C, Stanley S, Stapleton C, Stone SP (2015). Bridging the gap between pragmatic intervention design and theory: using behavioural science tools to modify an existing quality improvement programme to implement “Sepsis Six”. Implement Sci.

[40] Leis JA, Shojania KG (2017). A primer on PDSA: executing plan–do–study–act cycles in practice, not just in name. BMJ Qual Saf.

[41] Balasubramanian BA, Cohen DJ, Davis MM, Gunn R, Dickinson LM, Miller WL (2015). Learning evaluation: blending quality improvement and implementation research methods to study healthcare innovations. Implement Sci.

[42] Jacquez F, Vaughn LM, Wagner E (2013). Youth as partners, participants or passive recipients: a review of children and adolescents in community-based participatory research (CBPR). Am J Community Psychol.

[43] Vicari M, Oliveras C, Gleeson H, Hatane L, Cluver L. Meaningful engagement of adolescents and young people in national and local HIV programming. World Health Organization; 2019.

[44] Gleeson HS, Oliveras Rodriguez CA, Hatane L, Hart DT (2018). Ending AIDS by 2030: the importance of an interlinked approach and meaningful youth leadership. J Int AIDS Soc..

[45] Ozer EJ (2016). Youth-led participatory action research: developmental and equity perspectives. Adv Child Dev Behav.

[46] FMOH. Guidelines for young person’s participation in research and access to sexual and reproductive health services in Nigeria 2014. [Available from: https://www.popcouncil.org/uploads/pdfs/2014HIV_YoungPersonsSRH-Nigeria.pdf.

[47] Day S, Kapogiannis BG, Shah SK, Wilson EC, Ruel TD, Conserve DF (2020). Adolescent participation in HIV research: consortium experience in low and middle-income countries and scoping review. Lancet HIV.

